# Platelet-Rich Plasma One Week Prior to Hyaluronic Acid vs. Platelet-Rich Plasma Alone for the Treatment of Knee Osteoarthritis

**DOI:** 10.3390/biomedicines10112805

**Published:** 2022-11-04

**Authors:** Ashim Gupta

**Affiliations:** 1 Regenerative Orthopaedics, Noida 201301, UP, India; ashim6786@gmail.com; 2 Indian Stem Cell Study Group (ISCSG) Association, Lucknow 226010, UP, India; 3 Future Biologics, Lawrenceville, GA 30043, USA; 4 BioIntegrate, Lawrenceville, GA 30043, USA; 5 South Texas Orthopaedic Research Institute (STORI Inc.), Laredo, TX 78045, USA

Knee osteoarthritis (OA) is the most recognized form of OA, responsible for approximately 4/5th of the global burden of the OA [1]. The prevalence of knee OA has continued to increase over the last few decades with no indications of deceleration [1]. Its pathophysiology involves synovial tissue inflammation and articular cartilage deterioration, leading to debilitating pain and loss of function [2,3]. Currently knee OA is managed by utilizing conservative treatment modalities, both pharmacological (such as non-steroidal anti-inflammatory drugs (NSAIDs), opioids, and corticosteroids) and non-pharmacological (such as weight loss, diet control, physical therapy, and activity adjustment), and surgery (such as knee arthroplasty (especially in advanced stages of knee OA)) when conservative treatment options have failed [2,3]. These conventional modalities have contraindications and side effects, incessantly aiming to decrease pain instead of targeting the underlying pathology [2,3].

Recently, clinicians have embraced the utilization of intra-articular injection of platelet-rich plasma (PRP) and hyaluronic acid (HA), either individually or in combination, to treat patients suffering with knee OA. Published studies, including systematic reviews, have reported that both PRP and HA, utilized individually, lead to improved clinical outcomes in terms of mitigating pain and decelerating OA progression; however, the outcomes obtained after PRP administration were better compared to those of HA [4,5]. In addition, there are some recent studies that evaluated the feasibility of combining PRP and HA as a dual therapy and compared it with PRP-alone therapy. The results from these studies reported a significant reduction in Visual Analogue Scale (VAS), and improvement in Western Ontario and McMaster Universities Arthritis Index (WOMAC) and International Knee Documentation Committee (IKDC) scores in the PRP plus HA group compared to PRP alone up to 1 year post-injection [1,6]. The potential additive benefits of combining PRP with HA to treat knee OA can be attributed to their disparate biological mechanisms, i.e., the regenerative ability of PRP and rheological properties of HA, to augment the activity of signal molecules, including growth factors, cytokines, inflammatory molecules, and catabolic enzymes [7,8,9,10,11,12,13]. Nonetheless, there are insufficient studies to support the use of this combined approach mainly due to methodological constraints, including difference in the type of PRP formulated (platelet count and concentration compared to whole blood, presence or absence of WBC or RBC, use of activator, etc.). 

In this editorial, I focus on a recently published clinical trial by Wu et al. [14], titled “Efficacy of a Novel Intra-Articular Administration of Platelet-Rich Plasma One Week Prior to Hyaluronic Acid versus Platelet-Rich Plasma Alone in Knee Osteoarthritis: A Prospective, Randomized, Double-Blind, Controlled Trial”. In this prospective, randomized, double-blinded, controlled study, the authors investigated the effects of a PRP and HA combination therapy, with a unique injection protocol on pain, functional activity, balance, and the risk of falls in knee OA patients. A total of 46 patients with unilateral knee OA (Ahlback stage I–III) were enrolled in this study in line with the inclusion (50–75 years old, pain in affected knee for >6 months, and VAS ≥ 4) and exclusion criteria (intra-articular injections 6 months before the study, NSAIDs within a week of study, previous knee surgery, thrombocytopenia (platelet count < 150,000), platelet dysfunction, coagulopathy, known autoimmune diseases or rheumatoid arthritis, allergic to any contents of the HA or local anesthetics, significant effusion of the joint prior to injection, and incapability to undergo balance testing), and randomized 1:1 to receive either a single-dose intra-articular injection of HA (intervention group) or normal saline (control group) one week after a single-dose injection of leukocyte-poor PRP. These patients were assessed at baseline (prior to injection) and at 1, 3, 6, and 12 months post-injection using WOMAC, and static balance and the risk of falls assessed by Biodex Balance System SD (Biodex Medical Systems, Shirley, NY, USA). A total of 45 patients completed the study (1 patient in the intervention group dropped voluntarily). No adverse effects were reported throughout the duration of this study. Both groups demonstrated statistically significant improvements in WOMAC scores at all follow-up timepoints compared to the baseline. The intervention group displayed significantly higher improvements compared to the control group in WOMAC pain, stiffness, and total scores at most of the follow-up timepoints (except 1 month: WOMAC pain, stiffness, and total scores; 3 months: WOMAC stiffness scores; 6 months: WOMAC total scores). Additionally, a reduction (not significant) in WOMAC function scores was observed in the intervention group compared to the control group. Both groups also showed significant improvements for balance and risk of falls for most follow-up timepoints (except 3 months: balance-OSI; 3–12 months: balance-APSI; 1–12 months: balance-MLSI) compared to the baseline. Moreover, significantly better reductions in balance-OSI at 3–12 months and balance-MLSI at 3–6 months follow-up timepoints were reported in the intervention group compared to the control group. The intervention group also demonstrated a propensity (not significant) toward higher reductions in balance-APSI scores and risk of falls compared to the control group. The results from this study demonstrate that the intra-articular injection of PRP one week prior to HA provides better symptom relief and enhances static balance compared to PRP alone in knee OA patients. This study has few limitations, including a small sample size and lack of information related to platelet count and concentration of platelets compared to baseline whole blood. 

In addition to the above-mentioned shortcomings, one concern is the non-inclusion of a group involving the simultaneous injection of PRP plus HA, similar to what has been reported in the literature [1,5]. The study’s authors stated that there is the probability of a lower synergistic effect of simultaneous PRP plus HA administration due to the dilution of growth factors in the PRP and the viscoelasticity of HA. This was one of the rationales for the study authors to conduct their study investigating the efficacy of PRP and HA administered a week apart. Thus, I believe that this group (simultaneous injection of PRP plus HA) should have been included to determine whether PRP and HA should be injected simultaneously or one week apart. 

In summary, despite the limitations, I applaud the efforts of the authors as this study positively adds to the current literature that the administration of PRP plus HA is safe and justifies the need for high-powered, prospective, multi-center, double-blinded, randomized controlled trials to further establish the efficacy of PRP plus HA (injected simultaneously and/or one week apart) compared to PRP alone and to determine the optimal dosage of PRP and HA needed to attain the best therapeutic effect. As of 29 October 2022, there are no ongoing clinical trials registered on clinicaltrials.gov (search terms: “knee osteoarthritis” and “platelet-rich plasma” and “hyaluronic acid”) comparing the efficacy of PRP plus HA (either administered together or apart) vs. PRP alone. 
